# Susceptibility to and transmission of H5N1 and H7N1 highly pathogenic avian influenza viruses in bank voles (*Myodes glareolus*)

**DOI:** 10.1186/s13567-015-0184-1

**Published:** 2015-05-13

**Authors:** Aurora Romero Tejeda, Roberta Aiello, Angela Salomoni, Valeria Berton, Marta Vascellari, Giovanni Cattoli

**Affiliations:** Istituto Zooprofilattico Sperimentale delle Venezie (IZSVe), OIE/FAO and National Reference Laboratory for Newcastle Disease and Avian Influenza, OIE Collaborating Centre for Infectious Diseases at the Human-Animal Interface, Viale dell’Università 10, Legnaro, 35020 Padova, Italy; Histopathology Laboratory, IZSVe, Viale dell’Università 10, Legnaro, 35020 Padova, Italy

## Abstract

The study of influenza type A (IA) infections in wild mammals populations is a critical gap in our knowledge of how IA viruses evolve in novel hosts that could be in close contact with avian reservoir species and other wild animals. The aim of this study was to evaluate the susceptibility to infection, the nasal shedding and the transmissibility of the H7N1 and H5N1 highly pathogenic avian influenza (HPAI) viruses in the bank vole (*Myodes glareolus*), a wild rodent common throughout Europe and Asia. Two out of 24 H5N1-infected voles displayed evident respiratory distress, while H7N1-infected voles remained asymptomatic. Viable virus was isolated from nasal washes collected from animals infected with both HPAI viruses, and extra-pulmonary infection was confirmed in both experimental groups. Histopathological lesions were evident in the respiratory tract of infected animals, although immunohistochemistry positivity was only detected in lungs and trachea of two H7N1-infected voles. Both HPAI viruses were transmitted by direct contact, and seroconversion was confirmed in 50% and 12.5% of the asymptomatic sentinels in the H7N1 and H5N1 groups, respectively. Interestingly, viable virus was isolated from lungs and nasal washes collected from contact sentinels of both groups. The present study demonstrated that two non-rodent adapted HPAI viruses caused asymptomatic infection in bank voles, which shed high amounts of the viruses and were able to infect contact voles. Further investigations are needed to determine whether bank voles could be involved as silent hosts in the transmission of HPAI viruses to other mammals and domestic poultry.

## Introduction

Influenza type A (IA) viruses are subtyped on the basis of two surface proteins, the haemagglutinin (HA) and the neuraminidase (NA), which govern the viral lifecycle at cellular entry and the release of progeny virions. To date, 16 HA and 9 NA subtypes have been described and isolated from wild water birds, the known primary natural reservoir of IA viruses [1]. However, the identification of two novel influenza A-like viruses, the H17N10 and the H18N11 strains isolated from a little yellow-shouldered bat from Guatemala [2] and from a flat-faced fruit bat from Peru [3], respectively, has prompted attention towards new mammalian species as possible hosts or reservoirs of unknown IA viruses.

Although transmission of IA viruses from avian to mammalian hosts is considered to be a rare event, due to the complexity of the transmission pathways and the different interfaces connecting both populations [4,5], species-adapted influenza lineages of avian origin are already circulating in swine, horses, dogs and humans [6,7], independently from the avian reservoir. The first evidence of natural infection with avian origin IA viruses in wild mammals was reported in harbour seals in 1979–1980 [8,9]. Since then, evidence of natural IA-related infections has been reported in several wild mammalian species such as mink [10], stone marten [11], raccoon [12], feral cat [13], feral dog [14], captive tiger and leopard [15], captive Owston’s palm civet [16], and skunk [17]. Recently, the discovery that wild pikas can be naturally infected with HPAI H5N1 [18] and LPAI H9N2 [19] subtypes has extended the range of known terrestrial free-ranging mammals hosting IA viruses. Moreover, susceptibility to low pathogenic IA viruses has been demonstrated by experimental infection in live-trapped synanthropic species such as cottontail rabbits [20] and the striped skunk [21].

Although the susceptibility of some wild mammals to avian IA infections has been documented, the potential role of these species in the IA ecology and its relevance in public health have received limited attention [18,21-23]. Very little information is available on how wild birds can spread IA viruses to free-living wild mammals within their natural habitat [18], and whether virus adaptation to these hosts could represent an advantage for transmission to other species, such as terrestrial mammals, poultry and eventually humans [4,22]. In this regard, a small number of studies have identified the presence of mammalian wildlife (rodents and racoons) as a risk factor associated with the spread of IA viruses among commercial poultry and duck farms [24-26], and although an evident link between the presence of wild rodents and the occurrence of IA infections in poultry farms does not exist, rodent control is widely recommended as biosecurity measure to limit the spread of IA viruses within poultry flocks [27,28].

Synanthropic rodents are highly mobile in urban and rural areas, and may represent a risk for virus transmission within and among poultry farms [28]. In addition, they may be at a higher risk of co-infection with avian and mammalian IA strains, providing conditions for reassortment in nature [22]. Interestingly, seroconversion to IA has been demonstrated in wild-caught rodents during several low pathogenic avian influenza (LPAI) and HPAI outbreaks in poultry, although no virus isolation attempts from lungs or other collected organs have provided successful results [28-31]. Moreover, it has been demonstrated that LPAI viruses derived from wild birds are able to efficiently replicate in lungs of wild-caught house mice without prior adaptation, elucidating the possible role of this species in outbreak dynamics on poultry farms [28]. Remarkably, Achenbach et al. confirmed that recently-infected mallards had been able to transmit a LPAI H7N3 isolate to rats directly or through environmental contamination in an artificial barnyard [32].

In the present study, we evaluated the susceptibility to infection, the nasal shedding and the transmissibility of two HPAI isolates of H7N1 and H5N1 subtypes in the bank vole (*Myodes glareolus*), a wild rodent common throughout Europe and Asia, with the aim of elucidating the potential role of wild synanthropic populations of rodents in IA virus ecology.

## Materials and methods

### Viruses

The two HPAI isolates used for this study were H7N1 A/ostrich/Italy/2332/2000 (Os/H7N1) [GenBank: PB2:DQ991309, PB1:DQ991310, PA:DQ991311, HA:DQ991312, NP:DQ991313, NA:DQ991314, MA:DQ991315, NS: DQ991316], isolated during the Italian epidemic of 1999–2000 [33], and H5N1 A/turkey/Turkey/1/2005 (Tk/H5N1) [GenBank: PB2: EF619975, PB1: EF619976, PA: EF619979, HA: EF619980, NP: EF619977, NA: EF619973, MA: EF619978, NS: EF619974]. The two viruses were replicated in the allantoic cavity of 9- to 11-days old embryonated-specific pathogen-free (SPF) hen eggs according to OIE guidelines [34], and allantoic fluids were titrated to calculate the 50% Embryo Infectious Dose (EID_50_) using the Reed and Muench formula [35].

### Animals

Eighty five (male and female) bank voles (*Myodes glareolus*) of 4–6 weeks of age (13–16 g body weight) were obtained from a colony originally derived from the Istituto Superiore di Sanità (ISS, Rome, Italy). Prior to each experiment, all animals were left for acclimatization to the local environment for at least 1 week. Bank voles were housed in mouse cages with standard rodent feed and water *ad libitum*, and were weekly supplemented with grain seeds. Cages were prepared with a 3–4 cm bedding layer, and animals were provided with sufficient nesting material and environmental enrichments in order to express their species-specific behaviour (digging and burrowing). All in vivo experiments, approved by the IZSVe Ethics Committee (CE.IZSVE.24/2014) and authorized by the Italian Ministry of Health (Decree N.180/2011-B), were performed in containment facilities (BSL-3) and in accordance with the relevant legislation on the use of animals for scientific purposes [36].

### Study design

#### Serology

Prior to challenge, blood was collected from all voles (tail vein) and sera were tested by means of a competitive commercial anti-nucleoprotein (NP) influenza ELISA (ID-screen®, IDVet, Montpellier, France) and through the Haemagglutination Inhibition (HI) test according to the WHO procedure used for mammal sera [37], using the challenge viruses as antigens (naïve sera from animals of each experimental group were tested only with the corresponding challenge virus). The same tests were used to evaluate the seroconversion on convalescent sera.

#### Nasal shedding experiment

Since no preliminary information existed regarding the infectivity of HPAI viruses in bank voles, the challenge dose of each selected virus was established as ten times the 50% Mouse Lethal Dose (MLD_50_) as for previous experiments in BALB/c mice [38].

To evaluate the occurrence of nasal shedding, two groups of 12 male and female bank voles each (groups Os/H7N1 and Tk/H5N1) were inoculated intranasally, under light anesthesia (50 mg/kg ketamine and 4 mg/kg xylazine, intraperitoneally), with 50 μL of PBS-diluted allantoic fluid containing 10 times the MLD_50_ calculated in Balb/C mice, equivalent to 10^3.75^ and 10^4.4^ EID_50_/0.1 mL of the H7N1 and H5N1 viruses, respectively. Three voles were used as negative controls and were mock-infected with 50 μL of sterile phosphate-buffered saline (PBS)-diluted allantoic fluid.

On a daily basis, challenged animals were monitored for the onset of clinical signs. Animals reaching the humane endpoint (weight loss greater than 20% and/or severe depression and respiratory distress) were euthanized. On days 3, 5, 7 and 9 post-infection (pi), three subjects were randomly selected and euthanized to collect organs. Brain, lungs and trachea tissues were collected from each animal along with a nasal wash (using 400 μL of sterile PBS). On day 3 post-inoculation, all control voles were also euthanized for collection of samples. Brain and nasal washes were tested by quantitative real time reverse transcriptase polymerase chain reaction (qRRT-PCR), while whole lungs and trachea were paraffin-embedded for histopathological and immunohistochemical (IHC) examination. Virus isolation in SPF embryonated hen eggs was attempted from all qRRT-PCR positive samples.

#### Pathogenicity and contact transmission experiment

Two groups of 24 female voles each were used for the transmission experiments; each group was randomly selected after weaning and reared together in a proper size cage (2065 cm^2^ floor area). For each isolate, the experimental group included 12 infected voles (groups I-Os/H7N1 and I-Tk/H5N1) and 12 sentinels. The voles of each infected group were challenged by the intranasal route, as previously described, with 50 μL of PBS-diluted isolate. Twenty-four hours later, infected voles were moved into a clean cage hosting a group of 12 serologically naïve sentinels. Contemporaneously, 10 mock-infected voles were used as controls.

On a daily basis, all animals were observed for the onset of clinical signs and for mortality, and body weight was monitored on days 2, 4, 7 and 9 pi. Significance in body weight changes was calculated by the Student’s *t*-test, and a *P* value less than 0.05 (*p* < 0.05) was considered statistically significant. On days 3 and 4 pi, post-contact (pc) and post-inoculation, two animals per group (infected, sentinels and control, respectively) were randomly selected and euthanized to collect nasal washes and different organs including lungs, trachea, brain, spleen, kidney and intestine. On day 30 pi/29 pc, all remaining voles were humanly euthanized, and convalescent sera were collected to determine seroconversion.

#### RRT-PCR and qRRT-PCR

*Sample preparation*. One hundred milligrams of tissues were homogenized in 200 μL of lysis buffer of the commercial kit Nucleospin RNA (Macherey Nagel, Düren, Germany), using the TissueLyser II (Qiagen, Germantown, USA) and following a homogenization program of 30 Hz for 3 min. The homogenates were clarified by centrifugation and tested by means of RRT-PCR.

*RNA extraction*. Viral RNA was extracted from 100 μL of clarified supernatant from tissue samples or from nasal washes, using the commercial kit Nucleospin RNA II and following the manufacturer’s instructions. Viral RNA was eluted in 60 μL of RNAse-free water and stored at −80 °C after the addition of 1 μL of RNasin Plus RNase Inhibitor (Promega®, Madison, USA).

*One*-*Step RRT*-*PCR and qRRT*-*PCR*. The extracted RNA was amplified using the published primers and probe from Spackman et al. [39], targeting the conserved matrix (M) gene of the IA virus. Five microlitres of RNA were added to the reaction mixture, composed of 300 nM of forward and reverse primers (M25F and M124-R, respectively) and 100 nM of fluorescent label probe (M + 64). The amplification reaction was performed in a final volume of 25 μL using the commercial QuantiTect Multiplex RT-PCR kit (Qiagen, Hilden, Germany). The RRT-PCR was performed using the following protocol: 20 min at 50 °C and 15 min at 95 °C, followed by 40 cycles at 94 °C for 45 s and 60 °C for 45 s. According to the internal validation trails, the samples with a threshold cycle (Ct) higher than 35 were considered negative; however, all Ct values obtained for each sample were reported in the results.

The same one-step RRT-PCR protocol was used for the qRRT-PCR analysis. The synthetic RNA used as reference standard was created in vitro by means of the MegaScript® T7 kit (Invitrogen, CA, USA) from the H7N1 (A/Ostrich/Italy/2332/2000) and H5N1 (A/turkey/Turkey/1/2005) HPAI strains.

#### Virus isolation

Nasal washes (around 200 μL) were diluted in 400 μL of PBS containing antibiotics (10 000 IU/mL of penicillin, 10 mg/mL of streptomycin, 5000 UI/mL of nystatin and 250 mg/mL of gentamycin sulphate), while tissue samples (100 mg) were homogenized using sterile polypropylene micropestles (Sigma-Aldrich, Hamburg, Germany) in 700 μL of PBS containing antibiotics, and then clarified by centrifugation. Processed samples were propagated into the allantoic cavities of 9 to 11 days-old SPF embryonated hen eggs according to the OIE guidelines [34].

#### Histopathology and Immunohistochemistry

After 24 h, the lungs and trachea collected from the infected and mock-infected voles were fixed in 10% neutral phosphate-buffered formalin, paraffin-embedded, and stained with hematoxylin-and-eosin method for histopathological examination. The immunohistochemistry (IHC) was carried out by an automated immunostainer (Autostainer™ Link 48 Dako, Italy). Briefly, the antigen retrieval was performed with Proteinase K (Dako, Italy) for 3 min at room temperature (RT). Endogenous peroxidases were neutralized by incubating the sections with the EnVision FLEX Peroxidase-Blocking Reagent (Dako, Italy) for 10 min at RT. Sections were incubated with a primary monoclonal antibody against IA virus nucleoprotein (Clone 1331, BIODESIGN International, USA), applied at 1:500 dilution for 10 min at RT. The EnVision FLEX/HRP (Dako, Carpinteria, CA, USA) and the EnVision FLEX Substrate Buffer EnVision FLEX DAB+ were used as the detection system and chromogen, respectively. Sections were then counterstained with the EnVision FLEX Hematoxylin (Dako, Italy). The specificity of the immunostaining was verified by incubating some sections with PBS instead of the specific primary antibody.

## Results

### Nasal shedding experiment

#### Serology

All voles sampled prior to infection tested negative for IA virus by means of a commercial ELISA and HI tests, while convalescent sera were not collected due to the short duration of this experiment (9 days).

#### Clinical signs and mortality

None of the twelve H7N1-infected voles showed any clinical sign throughout the experiment. Only one out of twelve H5N1-infected voles started to display the clinical signs of the disease (mild depression) on day 2 pi, being humanely euthanized on day 7 pi (severe depression and respiratory distress). All mock-infected voles remained asymptomatic throughout the duration of the experiment.

#### qRRT-PCR

**Figure 1:**
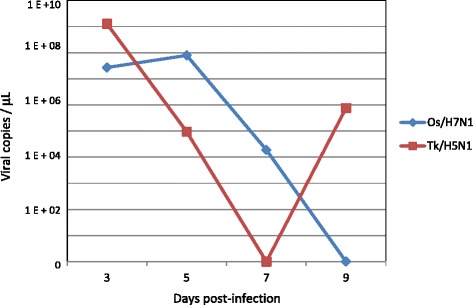
**Nasal shedding of infected voles at different time points of infection.** Mean of the viral shedding (viral copies/μL) recorded on nasal washes collected on days 3, 5, 7 and 9 pi from intranasally infected voles with 10^3.75^ EID_50_/0.1 mL of the Os/H7N1 strain (group Os/H7N1) and 10^4.4^ EID_50_/0.1 mL of the Tk/H5N1 strain (group Tk/H5N1).

Nasal shedding was detected in 1/3 voles of the Os/H7N1 group sampled on days 3 and 5 pi, showing 2.7 × and 7.9 × 10^7^ viral copies/μL, respectively. On day 7 pi, 1/3 nasal washes recorded a viral load of 1.89 × 10^4^ viral copies/μL (Figure 1). The nasal washes collected on day 9 pi, as well as all the brain samples collected from infected voles of this group at different points of infection, tested negative by qRRT-PCR. Results of nasal shedding of infected voles with both HPAI viruses at different time points of infection are reported in Table 1.

**Table 1:** **Results of the nasal shedding experiment**

**Day pi**	**H7N1 A/ostrich/Italy/2332/2000 (Os/H7N1)**				**H5N1 A/turkey/Turkey/1/2005 (Tk/H5N1)**				
	**Nasal wash**		**Lungs/trachea**		**Nasal wash**		**Brain**		**Lungs**
	**qRRT-PCR (Ct/SQ)**	**VI**	**HP**	**IHC**	**qRRT-PCR (Ct/SQ)**	**VI**	**qRRT-PCR (Ct/SQ)**	**VI**	**HP**
3	-	-	npl	+ trachea	+ (25.08/1.16 × 10^8^)	-	-	/	npl
	-	/	npl	-	+ (21.14/3.70 × 10^9^)	-	-	/	npl
	+ (24.0/2.7 × 10^7^)	-	Multifocal purulent and catarrhal bronchitis	+ lungs	+ (29.4/2.60 × 10^6^)	-	-	/	npl
5	+ (22.5/7.9 × 10^7^)	+	npl	-	-	/	-	/	Multifocal of PBP with lymphohistiocytic infiltrate
	-	/	npl	-	-	/	-	/	Multifocal of PBP with lymphohistiocytic infiltrate
	-	/	Multifocal alveolar haemorrhages	-	+ (33.18/9.40 × 10^4^)	-	-	/	Multifocal of interstitial pneumonia
7	-	/	npl	-	-	/	+(33.49/7.17 × 10^4^)^a^	-	Multifocal PBP
	-	/	Multifocal PBP	-	-	/	-	/	npl
	+ (34.19/1.89 × 10^4^)	-	Multifocal PBP	-	-	/	-	-	npl
9	-	/	Diffuse congestion	-	+ (34.68/2.50 × 10^4^)	-	-	-	Multifocal PBP
	-	/	npl	-	+ (30.03/1.50 × 10^6^)	-	-	/	npl
	-	/	npl	-	-	/	-	/	npl

Ct: quantification cycle; SQ: starting quantity (viral copies/μL); VI: virus isolation; HP: histopathological findings; IHC: immunohistochemical stain; −: negative; /: not performed; npl: no pathological lesions; PBP: pyogranulomatous bronchopneumonia, ^a^bank vole with clinical signs.

This Table shows the qRRT-PCR and virus isolation results of the nasal washes and brains collected from the voles intranasally infected with both HPAI strains at different time points of infection. The results of the histopathological findings and IHC examination of the lungs and trachea collected from the infected voles are also reported.

In H5N1-infected voles (Table 1), a peak of nasal shedding was recorded on day 3 pi, since 3/3 nasal washes tested positive with an average viral load of 1.27 × 10^9^ copies/μL (Figure 1). On day 5 pi, one out of three nasal washes collected tested positive, recording a viral load of 9.4 × 10^4^ viral copies/μL, while 2/3 nasal washes tested positive by qRRT-PCR on day 9 pi, with an average viral load of 7.62 × 10^5^ viral copies/μL (Figure 1). Interestingly, the brain sample collected on day 7 pi from the only symptomatic H5N1-infected vole tested positive with a viral load of 7.17 × 10^4^ copies/μL, while the remaining brain samples collected were negative.

All samples collected from the mock-infected voles were negative by means of qRRT-PCR.

#### Virus isolation

Viable virus was isolated from one qRRT-PCR positive nasal wash collected from one H7N1-infected vole on day 5 pi (Table 1), while no viable virus was recovered from any sample collected from H5N1-infected voles.

#### Histopathology and IHC

**Figure 2:**
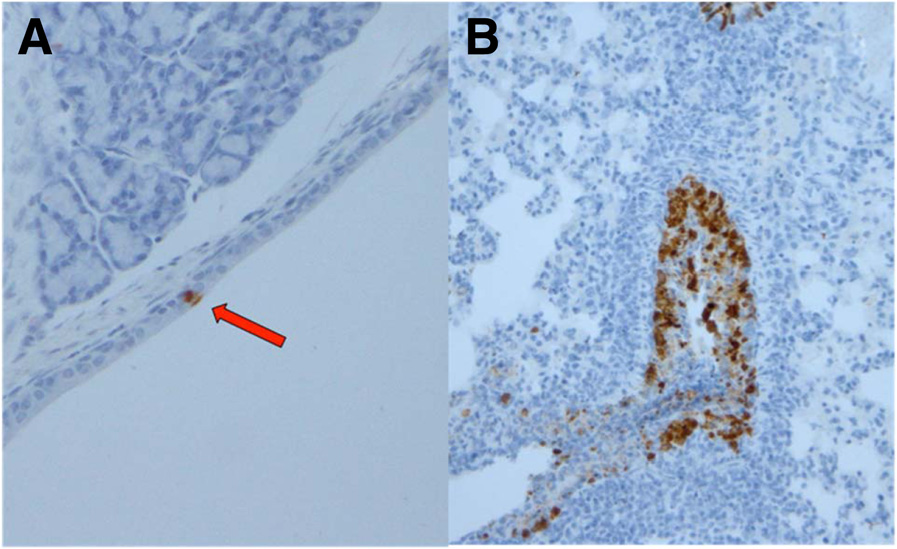
**Immunohistochemical detection of HPAI viruses in paraffin-embedded tissue sections from infected voles.** Positive staining by IHC in trachea **(A)** (red arrow) and lungs **(B)** collected on day 3 pi from two voles intranasally infected with 10^3.75^ EID_50_/0.1 mL of the Os/H7N1 virus.

Histopathological examination of lungs collected from H7N1-infected animals revealed lesions in 3/12 voles, mainly characterized by multifocal purulent and catarrhal bronchitis in one vole on day 3 pi, and by multifocal pyogranulomatous bronchopneumonia in two animals on day 7 pi (Table 1). Only two voles presented multifocal alveolar haemorrhages and diffuse pulmonary congestion on days 5 and 9 pi, respectively. No histopathological findings were observed in the tracheas collected from the H7N1-infected animals. Positive IHC staining was observed in the trachea and in the lungs sampled on day 3 pi from two different infected voles belonging to this group (Figure 2).

In the lungs collected from Tk/H5N1-infected voles, multifocal pyogranulomatous bronchopneumonia with lymphohistiocytic infiltrate was evident in 2/2 voles on day 5 pi, and in 1/3 voles on days 7 and 9 pi, respectively. Only one animal showed multifocal interstitial pneumonia on day 5 pi. In addition, none of the tracheas collected at different points of infection showed any pathological lesion. Despite the evident histopathological lesions observed in the lungs of 5/12 Tk/H5N1-infected voles, no positive IHC staining was detected in the lungs and tracheas evaluated in this experimental group. None of the mock-infected voles showed any significant histopathological lesions nor positivity to IHC staining in the lungs and trachea.

### Pathogenicity and by contact transmission experiment

#### Clinical signs and mortality

**Figure 3:**
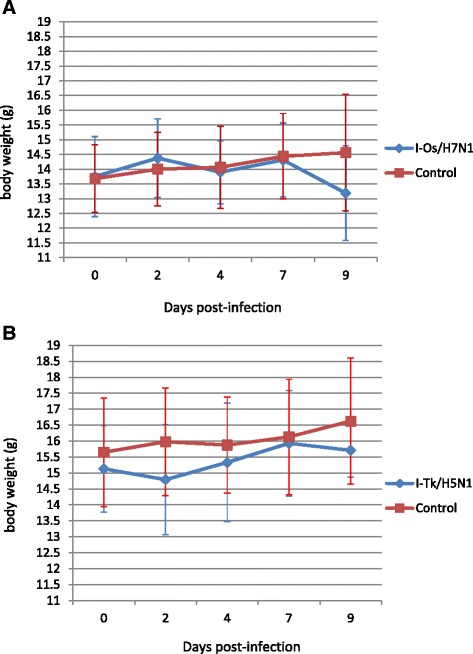
**Mean body weight changes of control voles and voles infected with HPAI viruses.** Body weight of bank voles intranasally inoculated with **(A)** 10^3.75^ EID_50_/0.1 mL of Os/H7N1 virus and **(B)** 10^4.4^ EID_50_/0.1 mL of Tk/H5N1 virus (group I-Tk/H5N1). Data shown are the mean ± SD (error bars) of body weight changes of inoculated voles in comparison to the control group. No significant difference (*p* > 0.05) was noticed between the body weight recorded at different points in all experimental groups.

None of the voles belonging to group I-Os/H7N1 showed any clinical sign within 30 days of the observation period, although mild reduction in body weight compared to the control group was observed on day 9 pi (Figure 3A). However, the difference in body weight loss at different days pi between both groups was not statistically significant (*p* > 0.05). Moreover, no clinical signs were observed in the sentinel group throughout the observation period.

One out of twelve H5N1-infected voles (group I-Tk/H5N1) showed depression, anorexia and severe respiratory signs on day 2 pi, and died during handling. No visual signs of disease were observed in the remaining infected voles and neither in all the contact sentinels; as well, the mean body weight recorded at different days pi showed no significant difference (*p* > 0.05) when compared to the control group (Figure 3B).

#### RRT-PCR

From the I-Os/H7N1 group, viral RNA was detected in all lungs collected on days 3 and 4 pi, and in 1/2 nasal washes collected on days 3 and 4 pi, respectively (Table 2). Moreover, viral RNA was detected in extra-respiratory organs such as the spleen (2/2), kidney (1/2) and brain (1/2) of infected and asymptomatic voles on day 3 pi. All samples collected from intestines tested negative by molecular test. From the contact sentinels of the Os/H7N1 group, all samples collected on day 3 pc tested negative, while one out of two lungs and one nasal wash collected on day 4 pc tested positive by RRT-PCR (Table 2).

**Table 2:** **Results of the pathogenicity and by contact transmission experiment**

**Virus**	**Sample**	**Day pi/pc**	**Infected**			**Sentinels**		
			**RRT-PCR**	**Ct ± SD**	**VI**	**RRT-PCR**	**Ct**	**VI**
Os/H7N1	Lungs	3	+2/2	26.22 ± 1.1	+2/2	-	-	nt
		4	+2/2	22.33 ± 0.08	+2/2	+1/2	28.24	+1/1
	Nasal wash	3	+1/2	32.13	**-**	-	-	nt
		4	+1/2	30.57	**-**	+1/2	35	-
	Brain	3	+1/2	30.01	**-**	-	-	nt
		4	-	-	nt	-	-	nt
	Spleen	3	+2/2	28 ± 3.5	+1/2	-	-	nt
		4	-	-	nt	-	-	nt
	Kidney	3	+1/2	29.6	-	-	-	nt
Tk/H5N1	Lungs	2^a^	+1/1	20.22	+1/1	nt	nt	nt
		3	+2/2	22.19 ± 2	+2/2	-	-	nt
		4	+2/2	27.3 ± 3.4	+2/2	+1/2	24.03	+1/1
	Nasal wash	2^a^	+1/1	26.95	-	nt	nt	nt
		3	+2/2	29 ± 2.1	+2/2	-	-	nt
		4	+2/2	34.7 ± 0.1	-	-	-	nt
	Brain	2^a^	-	-	nt	nt	nt	nt
		3	-	-	nt	-	-	nt
		4	+1/2	33.48	-	-	-	nt
	Spleen	2^a^	+1/1	33.12	-	nt	nt	nt
		3	-	-	nt	-	-	nt
		4	-	-	nt	-	-	nt

pi: post-infection; pc: post-contact; Ct: quantification cycle; SD: standard deviation; VI: virus isolation; ^a^dead vole with clinical signs on day 2 pi; −: all samples tested negative; nt: not tested.

RRT-PCR (mean of Ct values ± SD) and virus isolation results of the different organs and nasal washes collected from infected voles and in contact sentinels on days 3 and 4 pi/pc.

All lungs and nasal washes collected from H5N1-infected voles (group I-Tk/H5N1) were positive on days 3 and 4 pi. Moreover, one brain sample was positive in 1/2 voles on day 4 pi. Interestingly, the lungs, nasal wash and spleen collected from the only symptomatic H5N1-infected vole were positive by RRT-PCR on day 2 pi. From the contact sentinels of this group, the lungs collected from one animal on day 4 pc tested positive.

#### Virus isolation

Viable virus was isolated from all lungs collected on days 3 and 4 pi from infected voles of the group I-Os/H7N1 (Table 2). When virus isolation was attempted from other RRT-PCR positive organs, viable virus was isolated only from spleen on day 3 pi. Moreover, viable virus was isolated from the RRT-PCR positive lungs collected from the sentinel on day 4 pc.

As for the I-Tk/H5N1 group, viable virus was isolated from 5/5 RRT-PCR positive lungs collected from 2 infected voles on days 3 and 4 pi, respectively, and from the symptomatic vole that had died on day 2 pi (Table 2). All nasal washes collected on day 3 pi tested positive by VI. No virus isolation attempts were successful from other RRT-PCR positive extra-pulmonary organs, such as the brain and spleen collected from the infected group. Interestingly, viable virus was isolated from one RRT-PCR positive lung collected on day 4 pc from one in contact sentinel.

#### Serology

Six out of eight H7N1-infected voles (75%) tested positive by either competitive ELISA and HI tests on day 30 pi (Table 3). The HI titres ranged from 1:10 to 1:40. Four out of eight contact sentinels (50%) from the Os/H7N1 group were positive by ELISA and confirmed by HI test, with titres comparable to those of the infected voles (ranging from 1:10 to 1:40).

**Table 3:** **Results of seroconversion of the pathogenicity and contact transmission experiment**

**Virus**	**Inoculated**				**Sentinels**			
	**Clinical signs**	**Mortality**	**Seroconversion**		**Clinical signs**	**Mortality**	**Seroconversion**	
			**ELISA**	**HI**			**ELISA**	**HI**
**Os/H7N1**	Not observed	0% (0/8)	75% (6/8)	75% (6/8)^a^	Not observed	0% (0/8)	50% (4/8)	50% (4/8)^b^
**Tk/H5N1**	Severe respiratory signs (1/8)	12.5% (1/8)	100% (7/7)	100% (7/7)^c^	Not observed	0% (0/8)	12.5% (1/8)	0% (0/8)

^a^HI titers: 1:10 (*n* = 1), 1:20 (*n* = 3), 1:40 (*n* = 2); ^b^HI titers: 1:10 (*n* = 1), 1:20 (*n* = 1), 1:40, (*n* = 2); ^c^1:10 (*n* = 3), 1:20 (*n* = 4).

Clinical signs, mortality and seroconversion by means of ELISA and HI tests of inoculated and in contact sentinel voles on days 30 pi and 29 pc, respectively.

Regarding the I-Tk/H5N1 group, 7/7 infected voles (100%) seroconverted by both ELISA and HI tests, with HI titres ranging from 1:10 to 1:20. Only one out of eight (12.5%) contact sentinels tested positive by means of ELISA, but that individual was negative by HI.

## Discussion

The aim of this study was to investigate the susceptibility of the bank vole to infection with two HPAI strains, the H5N1 A/turkey/Turkey/1/2005 (Tk/H5N1) and the H7N1 A/ostrich/Italy/2332/2000 (Os/H7N1) isolates, and to establish whether those avian viruses are transmitted by infected animals to naïve co-housed sentinel voles.

In the nasal shedding experiment, no clinical signs were observed in any of the H7N1-infected voles, while only one vole infected with the H5N1 virus displayed severe clinical signs. Voles infected with both HPAI viruses demonstrated evident nasal shedding; however, the number of voles shedding virus (particularly on day 3 pi) was higher in the group of voles infected with the Tk/H5N1 virus in comparison to the animals infected with the Os/H7N1 strain (100% of shedders vs. 33%, respectively). Interestingly, Tk/H5N1-infected voles shed the virus for a longer period than those infected with Os/H7N1, since positivity in nasal washes was recorded until day 9 pi in this group (66.6% vs. 0% of shedders). Quantitatively, voles infected with the Tk/H5N1 virus shed a higher amount of viruses than voles infected with the Os/H7N1 strain, especially on days 3 and 9 pi (1.27 × 0^9^ vs. 2.7 × 10^7^ and 7.6 × 10^5^ vs. 0 viral copies/μL, respectively). Infected voles showed a similar shedding pattern to those reported in BALB/c mice infected with the same infectious doses of both HPAI isolates [38], although a different clinical outcome and mortality rate were observed in infected animals (100% mortality within 7 days pi of BALB/c mice infected with both strains vs. 0 and 8.3% of mortality in infected voles with Os/H7N1 and Tk/H5N1 strains, respectively). Viable virus was isolated from one nasal wash collected on day 5 pi from one Os/H7N1-infected vole, demonstrating that these animals are able to shed viable infectious particles to the environment. In contrast, no viable virus was recovered in the nasal washes collected from the Tk/H5N1-infected voles, despite their positivity by means of RRT-PCR. Due to the scarcity of original sample material, it was not possible to replicate the isolation attempts, which may explain the low virus isolation rate observed. However, the viable H5N1 virus was isolated in nasal washes collected in the pathogenicity and transmission experiment.

The histopathological examination of the respiratory tract of asymptomatic voles infected with both HPAI strains demonstrated the presence of lesions due to the influenza infection, mainly characterized by multifocal pyogranulomatous bronchopneumonia, in 25% (3/12) and 41% (5/12) of infected voles with Os/H7N1 and Tk/H5N1 viruses, respectively. However, IHC positivity was only detected in trachea and lungs of two Os/H7N1-infected voles on day 3 pi, confirming the occurrence of viral replication in the upper and lower respiratory tract for this strain. Although no IHC positivity was observed in any organ collected from Tk/H5N1-infected animals, the positivity obtained by RRT-PCR on nasal washes in this same group suggests that further studies of the upper respiratory tract of voles (especially nasal mucosa and turbinates) are needed.

As observed in the nasal shedding experiment, all voles infected with the Os/H7N1 virus in the transmission experiment did not show any clinical sign, while only one vole infected with Tk/H5N1 displayed several respiratory signs and died on day 2 pi. Moreover, both infected groups showed no significant difference in body weight variations compared to the control group at different time points of infection. Despite the asymptomatic infection, the two selected HPAI viruses spread systemically in bank voles, since viral RNA from both of them was detected in extra-pulmonary organs, such as brain, kidney and spleen. Interestingly, as observed in the nasal shedding experiment, viral RNA was found in the nasal washes collected in a higher number of animals infected with the Tk/H5N1 virus than in those infected with the Os/H7N1 strain. Although the oral-fecal route of transmission could not be ruled out in this experiment, these findings suggest that the respiratory route may be the predominant mode of transmission of both HPAI viruses in this species. In fact, the viable virus was isolated from the lungs and nasal washes of infected voles, while no evidence of positivity and/or viral replication was observed in any of the intestines collected. However, further experiments using the oral route of inoculation will be necessary to clarify whether influenza viruses are able to replicate in the gastrointestinal tract of voles, and if infected animals are able to shed viruses to the environment by means of the feaces.

Importantly, voles infected with both HPAI viruses were able to infect in contact voles. No clinical signs of disease were observed in any of the sentinels, although virological (lungs and nasal washes) positivity was confirmed on day 4 pc in 2 and 1 sentinels from the Os/H7N1 and Tk/H5N1 groups, respectively. Although both the selected HPAI strains harboured the PB2 E627K mutation; which is correlated with higher pathogenicity, adaptation and transmission in mammals [40,41]; the Os/H7N1 virus displayed a higher rate of transmission than the Tk/H5N1 virus, since seroconversion was confirmed in 50% (4/8) and 12.5% (1/8) of sentinels at the end of the experiment, respectively.

Although our experimental design did not include the sampling of nasal washes on day 1 pi, it was demonstrated that both LPAI and HPAI viruses replicate in the upper respiratory tract (in particular, in the nasal turbinates) of mice [28,42,43] and other mammals [21,44] as early as day 1 pi. Moreover, we also excluded the possibility that the natural infection of sentinels was due to residual inoculums in the nasal cavity of the inoculated voles, since sentinels were put in contact with the infected animals after the recommended period of 6 h post inoculation to avoid any contamination [45].

The present study demonstrated that the bank vole is susceptible to infection with highly pathogenic H5N1 and H7N1 viruses of avian origin without prior adaptation, shedding viable virus in the environment, and transmitting the virus to contact voles. Interestingly, infection with the same HPAI strains demonstrated high pathogenicity and 100% of mortality in BALB/c mice [38], while infected voles were more resistant to the clinical condition and displayed zero or very low mortality rate. These important findings may suggest that wild rodents could play a role as silent hosts in IA virus epidemiology, contributing to the spread of HPAI virus infections during an outbreak. If this occurs under natural conditions, the circulation of IA viruses in these rodents may provide opportunities for the acquisition of mammalian adaptive mutations, which could minimize the barriers to interspecies transmission of these viruses. However, the study of synanthropic wild mammals, and in particular wild rodents, is a critical and important gap in our knowledge of how IA viruses may evolve in new hosts [22], considering that these species share the same ecological habitats as waterfowl and live commensally around domestic poultry farms [32], thus running the risk of being exposed to IA viruses.

Bank voles are geographically distributed in central Europe, in the South to Northern Spain and Italy, the Balkans, Western and Northern Turkey, as well as in Britain and Ireland [46], sharing a wide variety of habitats (woodland, river and stream banks, scrub and parkland) with the wild reservoirs of all avian IA strains and other terrestrial mammals. Importantly, voles are well identified as natural hosts for viruses of zoonotic interest, such as hantavirus Puumala virus [47], flavivirus tick borne encephalitis virus [48], orthopoxvirus cowpox virus [49], and parechovirus Ljungan virus [50]. The transmission of most of these viruses from rodents to humans is believed to occur through inhalation of aerosols contaminated by viruses shed in excreta, saliva and urine of infected animals; and several outdoor activities, such as camping and cleaning rodent infestations are considered important risk factors associated with their transmission [51]. In addition, multiple infections in voles with zoonotic pathogens such as *Borrelia* and *Bartonella* [52], as well as the presence of other pathogens as *Leptospira*, *Babesia* and *Anaplasma* have been reported [53]. All these findings demonstrate that voles are potentially susceptible to be infected by several pathogens with zoonotic potential, which could be transmitted directly or indirectly to other wild rodents and mammals.

Further investigations are needed to address the virological presence and the natural seroprevalence of IA viruses in free-living bank voles and other wild rodents, in the hope that a more conclusive epidemiologic scenario of IA infections in these synanthropic species may be provided, especially when avian and wild mammalians populations overlap in nature [22]. In addition, it would be important to understand whether the infection with IA viruses in synanthropic mammalian species may have a substantial impact on the public health, and if these species could be involved in the IA transmission to other synantropic populations of rodents, domestic poultry and mammals.

Two non-rodent adapted HPAI viruses caused asymptomatic infection in bank voles, which shed viable infectious particles capable of infecting in-contact sentinels. Infection of BALB/c mice with the same strains in previous experiments caused 100% mortality and severe clinical signs, thus suggesting that voles are more resistant to the disease caused by HPAI viruses, but still potentially capable of transmitting the virus. Additional studies are needed to elucidate the potential role of wild rodents in the epidemiology of HPAI infections.

## Abbreviations

IA: Influenza type A
HPAI: Highly pathogenic avian influenza
LPAI: Low pathogenic avian influenza
HA: Haemagglutinin
NA: Neuraminidase
NP: Nucleoprotein
SPF: Specific pathogen-free
EID_50_: 50 percent Embryo Infectious Dose
MLD_50_: 50 percent Mouse Lethal Dose
PBS: Phosphate-buffered saline
HI: Haemagglutination inhibition test
pi: Post-infection
pc: Post-contact
RRT-PCR: Real time reverse transcription polymerase chain reaction
qRRT-PCR: Quantitative RRT-PCR
Ct: Quantification cycle
HP: Histopathological
IHC: Immunohistochemical stain
VI: Virus isolation
PBP: Pyogranulomatous bronchopneumonia

## References

[1] Webster RG, Bean WJ, Gorman OT, Chambers TM, Kawaoka Y (1992). Evolution and ecology of influenza A viruses. Microbiol Rev.

[2] Tong S, Li Y, Rivailler P, Conrardy C, Castillo DA, Chen LM, Recuenco S, Ellison JA, Davis CT, York IA, Turmelle AS, Moran D, Rogers S, Shi M, Tao Y, Weil MR, Tang K, Rowe LA, Sammons S, Xu X, Frace M, Lindblade KA, Cox NJ, Anderson LJ, Rupprecht CE, Donis RO (2012). A distinct lineage of influenza A virus from bats. Proc Natl Acad Sci U S A.

[3] Tong S, Zhu X, Li Y, Shi M, Zhang J, Bourgeois M, Yang H, Chen X, Recuenco S, Gomez J, Chen LM, Johnson A, Tao Y, Dreyfus C, Yu W, McBride R, Carney PJ, Gilbert AT, Chang J, Guo Z, Davis CT, Paulson JC, Stevens J, Rupprecht CE, Holmes EC, Wilson IA, Donis RO (2013). New world bats harbor diverse influenza A viruses. PLoS Pathog.

[4] Reperant LA, Kuiken T, Osterhaus AD (2012). Adaptive pathways of zoonotic influenza viruses: from exposure to establishment in humans. Vaccine.

[5] Dórea FC, Cole DJ, Stallknecht DE (2013). Quantitative exposure assessment of waterfowl hunters to avian influenza viruses. Epidemiol Infect.

[6] Harder TC, Siebert U, Wohlsein P, Vahlenkamp T (2013). Influenza A virus infections in marine mammals and terrestrial carnivores. Berl Munch Tierarztl Wochenschr.

[7] Reperant LA, Rimmelzwaan GF, Kuiken T (2009). Avian influenza viruses in mammals. Rev Sci Tech.

[8] Geraci JR, St Aubin DJ, Barker IK, Webster RG, Hinshaw VS, Bean WJ, Ruhnke HL, Prescott JH, Early G, Baker AS, Madoff S, Schooley RT (1982). Mass mortality of harbor seals: pneumonia associated with influenza A virus. Science.

[9] Webster RG, Hinshaw VS, Bean WJ, Van Wyke KL, Geraci JR, St Aubin DJ, Petursson G (1981). Characterization of an influenza A virus from seals. Virology.

[10] Yoon KJ, Schwartz K, Sun D, Zhang J, Hildebrandt H (2012). Naturally occurring Influenza A virus subtype H1N2 infection in a Midwest United States mink (*Mustela vison*) ranch. J Vet Diagn Invest.

[11] Klopfleisch R, Wolf PU, Wolf C, Harder T, Starick E, Niebuhr M, Mettenleiter TC, Teifke JP (2007). Encephalitis in a stone marten (*Martes foina*) after natural infection with highly pathogenic avian influenza virus subtype H5N1. J Comp Pathol.

[12] Horimoto T, Maeda K, Murakami S, Kiso M, Iwatsuki-Horimoto K, Sashika M, Ito T, Suzuki K, Yokoyama M, Kawaoka Y (2011). Highly pathogenic avian influenza virus infection in feral raccoons. Japan. Emerg Infect Dis.

[13] Gordy JT, Jones CA, Rue J, Crawford PC, Levy JK, Stallknecht DE, Tripp RA, Tompkins SM (2012). Surveillance of feral cats for influenza A virus in north central Florida. Influenza Other Respir Viruses.

[14] Su S, Zhou P, Fu X, Wang L, Hong M, Lu G, Sun L, Qi W, Ning Z, Jia K, Yuan Z, Wang H, Ke C, Wu J, Zhang G, Gray GC, Li S (2014). Virological and epidemiological evidence of avian influenza virus infections among feral dogs in live poultry markets, china: a threat to human health?. Clin Infect Dis.

[15] Keawcharoen J, Oraveerakul K, Kuiken T, Fouchier RA, Amonsin A, Payungporn S, Noppornpanth S, Wattanodorn S, Theambooniers A, Tantilertcharoen R, Pattanarangsan R, Arya N, Ratanakorn P, Osterhaus DM, Poovorawan Y (2004). Avian influenza H5N1 in tigers and leopards. Emerg Infect Dis.

[16] Roberton SI, Bell DJ, Smith GJ, Nicholls JM, Chan KH, Nguyen DT, Tran PQ, Streicher U, Poon LL, Chen H, Horby P, Guardo M, Guan Y, Peiris JS (2006). Avian influenza H5N1 in viverrids: implications for wildlife health and conservation. Proc Biol Sci.

[17] Britton AP, Sojonky KR, Scouras AP, Bidulka JJ (2010). Pandemic (H1N1) 2009 in skunks, Canada. Emerg Infect Dis.

[18] Zhou J, Sun W, Wang J, Guo J, Yin W, Wu N, Li L, Yan Y, Liao M, Huang Y, Luo K, Jiang X, Chen H (2009). Characterization of the H5N1 highly pathogenic avian influenza virus derived from wild pikas in China. J Virol.

[19] Yu Z, Cheng K, Sun W, Xin Y, Cai J, Ma R, Zhao Q, Li L, Huang J, Sang X, Li X, Zhang K, Wang T, Qin C, Qian J, Gao Y, Xia X (2014). Lowly pathogenic avian influenza (H9N2) infection in Plateau pika (Ochotona curzoniae), Qinghai Lake, China. Vet Microbiol.

[20] Root JJ, Shriner SA, Bentler KT, Gidlewski T, Mooers NL, Spraker TR, VanDalen KK, Sullivan HJ, Franklin AB (2014). Shedding of a low pathogenic avian influenza virus in a common synanthropic mammal–the cottontail rabbit. PLoS One.

[21] Root JJ, Shriner SA, Bentler KT, Gidlewski T, Mooers NL, Ellis JW, Spraker TR, VanDalen KK, Sullivan HJ, Franklin AB (2014). Extended viral shedding of a low pathogenic avian influenza virus by striped skunks (*Mephitis mephitis*). PLoS One.

[22] Runstadler J, Hill N, Hussein IT, Puryear W, Keogh M (2013). Connecting the study of wild influenza with the potential for pandemic disease. Infect Genet Evol.

[23] Vandalen KK, Shriner SA, Sullivan HJ, Rooot JJ, Franklin AB (2009). Monitoring exposure to avian influenza viruses in wild mammals. Mammal Rev.

[24] Duvauchelle A, Huneau-Salaün A, Balaine L, Rose N, Michel V (2013). Risk factors for the introduction of avian influenza virus in breeder duck flocks during the first 24 weeks of laying. Avian Pathol.

[25] Wakawa AM, Abdu PA, Oladele SB, Sa’idu L, Mohammed SB (2012). Risk factors for the occurrence and spread of Higlhy Pathogenic Avian Influenza H5N1 in commercial poultry farms in Kano, Nigeria. Sokoto J of Vet Sci.

[26] McQuiston JH, Garber LP, Porter-Spalding BA, Hahn JW, Pierson FW, Wainwright SH, Senne DA, Brignole TJ, Akey BL, Holt TJ (2005). Evaluation of risk factors for the spread of low pathogenicity H7N2 avian influenza virus among commercial poultry farms. J Am Vet Med Assoc.

[27] Koch G, Elbers ARW (2005). Outdoor ranging of poultry: a major risk factor for the introduction and development of High-Pathogenicity Avian Influenza. NJAS.

[28] Shriner SA, Vandalen KK, Mooers NL, Ellis JW, Sullivan HJ, Root JJ, Pelzel AM, Franklin AB (2012). Low-pathogenic avian influenza viruses in wild house mice. PLoS One.

[29] Nettles VF, Wood JM, Webster RG (1985). Wildlife surveillance associated with an outbreak of lethal H5N2 avian influenza in domestic poultry. Avian Dis.

[30] Shortridge KF, Gao P, Guan Y, Ito T, Kawaoka Y, Markwell D, Takada A, Webster RG (2000). Interspecies transmission of influenza viruses: H5N1 virus and a Hong Kong SAR perspective. Vet Microbiol.

[31] Henzler DJ, Kradel DC, Davison S, Ziegler AF, Singletary D, DeBok P, Castro AE, Lu H, Eckroade R, Swayne D, Lagoda W, Schmucker B, Nesselrodt A (2003). Epidemiology, production losses, and control measures associated with an outbreak of avian influenza subtype H7N2 in Pennsylvania (1996–98). Avian Dis.

[32] Achenbach JE, Bowen RA (2011). Transmission of avian influenza A viruses among species in an artificial barnyard. PLoS One.

[33] Capua I, Mutinelli F, Bozza MA, Terregino C, Cattoli G (2000). Highly pathogenic avian influenza (H7N1) in ostriches (*Struthio camelus*). Avian Pathol.

[34] OIE Manual of Diagnostic Tests and Vaccines for Terrestrial Animals 2014. http://www.oie.int/fileadmin/Home/eng/Health_standards/tahm/2.03.04_AI.pdf. Accessed 15 December 2014.

[35] Reed LJ, Muench H (1938). A simple method of estimating fifty per cent endpoints. Am J of Hyg.

[36] Directive 2010/63/EU of the European Parliament and of the Council of 22 September 2010 on the protection of animals used for the scientific purposes. http://eur-lex.europa.eu/legal-content/EN/TXT/?qid=1414768135290&uri=CELEX:32010L0063. Accessed 15 December 2014.

[37] WHO Manual on Animal Influenza Diagnosis and Surveillance. http://www.who.int/csr/resources/publications/influenza/en/whocdscsrncs20025rev.pdf Accessed 15 December 2014.

[38] Rigoni M, Toffan A, Viale E, Mancin M, Cilloni F, Bertoli E, Salomoni A, Marciano S, Milani A, Zecchin B, Capua I, Cattoli G (2010). The mouse model is suitable for the study of viral factors governing transmission and pathogenesis of highly pathogenic avian influenza (HPAI) viruses in mammals. Vet Res.

[39] Spackman E, Senne DA, Myers TJ, Bulaga LL, Garber LP, Perdue ML, Lohman K, Daum LT, Suarez DL (2002). Development of a real-time reverse transcriptase PCR assay for type A influenza virus and the avian H5 and H7 hemagglutinin subtypes. J Clin Microbiol.

[40] Zhang H, Li X, Guo J, Li L, Chang C, Li Y, Bian C, Xu K, Chen H, Sun B (2014). The PB2 E627K mutation contributes to the high polymerase activity and enhanced replication of H7N9 influenza virus. J Gen Virol.

[41] de Jong RM, Stockhofe-Zurwieden N, Verheij ES, de Boer-Luijtze EA, Ruiter SJ, de Leeuw OS, Cornelissen LA (2013). Rapid emergence of a virulent PB2 E627K variant during adaptation of highly pathogenic avian influenza H7N7 virus to mice. Virol J.

[42] Whiteley A, Major D, Legastelois I, Campitelli L, Donatelli I, Thompson CI, Zambon MC, Wood JM, Barclay WS (2007). Generation of candidate human influenza vaccine strains in cell culture - rehearsing the European response to an H7N1 pandemic threat. Influenza Other Respir Viruses.

[43] Cox RJ, Major D, Hauge S, Madhun AS, Brokstad KA, Kuhne M, Smith J, Vogel FR, Zambon M, Haaheim LR, Wood J (2009). A cell-based H7N1 split influenza virion vaccine confers protection in mouse and ferret challenge models. Influenza Other Respir Viruses.

[44] Hinshaw VS, Webster RG, Easterday BC, Bean WJ (1981). Replication of avian influenza A viruses in mammals. Infect Immun.

[45] Edenborough KM, Gilbertson BP, Brown LE (2012). A mouse model for the study of contact-dependent transmission of influenza A virus and the factors that govern transmissibility. J Virol.

[46] Wilson DE and Reeder DM (2005) Mammal Species of the World (MSW3), 3rd Edition. http://www.vertebrates.si.edu/msw/mswcfapp/msw/taxon_browser.cfm?msw_id=4452. Accessed 15 December 2014.

[47] Tersago K, Crespin L, Verhagen R, Leirs H (2012). Impact of Puumala virus infection on maturation and survival in bank voles: a capture/mark/recapture analysis. J Wildl Dis.

[48] Knap N, Korva M, Dolinšek V, Sekirnik M, Trilar T, Avšič-Županc T (2012). Patterns of tick/borne encephalitis virus infection in rodents in Slovenia. Vector Borne Zoonotic Dis.

[49] Chantrey J, Meyer H, Baxby D, Begon M, Bown KJ, Hazel SM, Jones T, Montgomery WI, Bennett M (1999). Cowpox: reservoir hosts and geographic range. Epidemiol Infect.

[50] Hauffe HC, Niklasson B, Olsson T, Bianchi A, Rizzoli A, Klitz W (2010). Ljungan virus detected in bank voles (*Myodes glareolus*) and yellow-necked mice (*Apodemus flavicollis*) from Northern Italy. J Wildl Dis.

[51] Winter CH, Brockmann SO, Piechotowski I, Alpers K, Van der Heiden M, Koch J, Stark K, Pfaff G (2009) Survey and case–control study during epidemics of Puumala virus infection. Epidemiol Infect 137:1479–1485. 10.1017/S0950268809002271 19288958

[52] Buffet JP, Marsot M, Vaumourin E, Gasqui P, Masséglia S, Marcheteau E, Huet D, Chapuis JL, Pisanu B, Ferquel E, Halos L, Vourc’h G, Vayssier-Taussat M (2012). Co-infection of *Borrelia afzelii* and *Bartonella spp*. in bank voles from a suburban forest. Comp Immunol Microbiol Infect Dis.

[53] Kallio ER, Begon M, Birtles RJ, Bown KJ, Koskela E, Mappes T, Watts PC (2014). First report of Anaplasma phagocytophilum and Babesia microti in rodents in Finland. Vector Borne Zoonotic Dis.

